# Software Sensor for Airflow Modulation and Noise Detection by Cyclostationary Tools

**DOI:** 10.3390/s20082414

**Published:** 2020-04-23

**Authors:** Mohamad Alkoussa Dit Albacha, Laurent Rambault, Anas Sakout, Kamel Abed Meraim, Erik Etien, Thierry Doget, Sebastien Cauet

**Affiliations:** 1 Laboratoire d’Informatique et Automatique des Systemes, University of Poitiers, 75016 Poitiers, France; mohamad.alkoussa.dit.albacha@univ-poitiers.fr (M.A.D.A.); erik.etien@univ-poitiers.fr (E.E.); thierry.doget@univ-poitiers.fr (T.D.); sebastien.cauet@univ-poitiers.fr (S.C.); 2 LaSIE Laboratory UMR CNRS 7356, 75016 la Rochelle, France; asakout@univ-lr.fr (A.S.); kamel.abed-meraim@univ-lr.fr (K.A.M.)

**Keywords:** acoustic measurements, fluid flow measurements, turbulent machine, cyclostationarity, software sensor

## Abstract

The paper presents tools to model low speed airflow coming from a turbulent machine. This low speed flow have instabilities who generate noise disturbances in the environment. The aim of the study proposed in this paper, is the using of cyclostationary tools with audio signals to model this airflow and detect the noisy frequencies to eliminate this noise. This paper also deals with the extraction in real time of the frequency corresponding to the noise nuisance. This extraction makes it possible to build a software sensor. This software sensor can be used to estimate the air flow rate and also to control a future actuator which will reduce the intensity of the noise nuisance. This paper focuses on the characteristic of the sound signal (property of cyclostationarity) and on the development of a software sensor. The results are established using an experimental setup representative of the physical phenomenon to be characterised.

## 1. Introduction

Signal processing is one of the most studied domains in the last decades. In the practical cases, most signals are not stationary and they are time dependent. Since non-stationary is a non-property, i.e there is no universal analysis tools in this case and we have to do the treatments separately for each case. In the late of 1950s, W.R. Bennett (1958) in [1], saw that there is a type of non-stationary signals that have specific characteristics like hidden periodicity in their structures. Based on these characteristics, he introduced a new concept as an extension of the stationarity or as a special case of the non-stationarity which is “cyclo-stationarity”. Then, in the 80s, this concept was taken up by Prof. William A. Gardner with several applications in the telecommunication field [2,3].

In the last years, the cyclo-stationary tools played a pivotal part in the signal processing domain. It helped to improve monitoring, diagnosis and characterisation of systems in the rotating machine and telecommunication fields. Very recently several publications discuss the use of cyclostationarity in different technological fields [4,5]. In this paper we will be using these cyclostationary tools for the first time for turbulent machines. S.Tardu discussed in [6] the implications of the cyclostationarity for the characterisation of turbulent flow, and the link between turbulent unsteady flow with imposed periodicity and cyclostationary process.

The aim of this paper is to contribute in the realisation of a software sensor that detects the fluid flow coming from a turbulent machine, than study this signal by the signal processing tools to model it. In each flow system we have a noisy part, and there are some works on removing this noise [7,8]. The original part in our paper is the using of the cyclo-stationary methods and the audio signals with the flow systems.

In fact to design an active control that eliminates the sound perturbation, it is important to analyse the mathematical and physical properties of the measured sound signal. On the other hand, in an industrial application, the noise disturbance have to be measured in real time. The entire device: acquisition of the sound signal and signal processing in real time to extract the useful information, constitutes a software sensor. The work presented in this paper deals with the analysis of the mathematical properties of the signal. Thus, a link can be made between the physical meaning of the sound signal and the useful part of the signal.

Hence, based on these methods, we can find the noisy frequencies, when each frequency has a physical realisation, then we can improve or eliminate the source of noise.

For a good treatment of the signals, we have to study the signal characteristics. We already know that flow systems aren’t stationary, that’s why we chose moving to the non-stationary tools and more precisely the cyclo-stationary ones. The comparison of our results by those of Jana HAMDI in [9] and Sofiane Maiz in [10] gives interesting results and made it possible to model these signals more clearly.

Section 2 presents some definitions, properties and extensions of the cyclo-stationary.

## 2. Cyclo-stationary: Definitions, Properties and Extensions

As a definition of the cyclo-stationary signal X(t), it is a signal that has a hidden periodicity in its structure. We can distinguish different orders of cyclo-stationarity.

The first order cyclo-stationarity is when a signal’s mean (moment of order 1) is periodic in time. i.e.,

(1) $$E\left\{X\left(t\right)\right\}=m_{x}\left(t\right)=m_{x}\left(t,T\right)$$

when T is the “cyclic period”.

The auto-correlation function (moment of order 2) is the dependence measurement between two different instants $t_{1}$ and $t_{2}$ (or *t* and t+τ). It is noted by $R_{x}\left(t_{1},t_{2}\right)$ (or $R_{x}\left(t,t+\tau \right)$) as the following:

(2) $$R_{x}\left(t_{1},t_{2}\right)=E\left\{X\left(t_{1}\right)X\left(t_{2}\right)\right\}$$

or,

(3) $$R_{x}\left(t,\tau \right)=E\left\{X\left(t\right)X\left(t+\tau \right)\right\}$$

(4) $$R_{x}\left(t,\tau \right)=E\left\{X\left(t+\tau /2\right)X\left(t-\tau /2\right)\right\}$$

When τ is equal to $t_{1}-t_{2}$. A signal who its auto-correlation function (moment of order 2) is periodic in time, it is called a second order cyclo-stationary signal. i.e.,

(5) $$E\left\{X\left(t\right)X\left(t+\tau \right)\right\}=R_{x}\left(t,\tau \right)=R_{x}\left(t+T,\tau \right)$$

In a more general way, we can say that a signal is cyclo-stationary of order “n”, when its moment of order “n” is periodic.

When we have first order cyclo-stationary and second order cyclo-stationary in the same time, it results the “Wide-sense cyclo-stationary”.

There are some extensions for the cyclo-stationarity cited by J.Antoni in [11] like “Pure and Impure cyclo-stationarity”, “poly-cyclostationarity” and “quasi-cyclostationary”, and others cited in [12,13] by F.Bonnardot like “Semi-cyclostationarity” and “Fuzzy Cyclostationarity”.

The most recent branch of cyclostationarity is the “cyclo-non-stationarity” that is introduced in 2013 by J. Antoni in [14], and it cited as a solution of the “wide-speed variation” and the “run-up” problems.

Normally, when we study the cyclostationarity, we have to show our signal in the frequency domain. Hence, the calculation of the power spectral density (PSD) is very important. There are two ways to calculate the PSD. For the first method, the first step is applying the Fourier transform to the auto-correlation function compared to τ. We obtain then the instantaneous spectrum or “Wigner-Ville spectrum” $W_{x}\left(t,f\right)$. The second step is given by applying the Fourier series to this instantaneous spectrum compared to *t* to obtain the “cyclic power spectra” $S_{x}^{\alpha }\left(f\right)$ or the PSD $S_{x}\left(\alpha ,f\right)$ when α=K/T is the cyclic frequencies and K∈Z [10,15,16]. If X(t) is stationary then we apply this method with α=0.

The second way has also two steps. The first one is to calculate the “cyclic auto-correlation function” $R_{x}^{\alpha }\left(\tau \right)$ by applying the Fourier series compared to *t*. then we apply the Fourier transform compared to τ as the second step to obtain $S_{x}^{\alpha }\left(f\right)$ or $S_{x}\left(\alpha ,f\right)$ [16].

Another function also very important in our work is the “cyclic spectral coherence” (SCoh). A cyclostationary signal has correlations in its spectral components spaced apart by the cyclic frequencies α. The strength of these correlations is measured by the cyclic coherence function [16]. It is defined as

(6) $$\gamma_{x}^{\alpha }\left(f\right)=\frac{S_{x}^{\alpha }\left(f\right)}{\sqrt{S_{x}^{0}\left(f+\alpha /2\right)S_{x}^{0}\left(f-\alpha /2\right)}}$$

with $0⩽|\gamma_{x}^{\alpha }\left(f\right)|⩽1$.

In the Section 5 we will be using the power spectral density PSD and the cyclic spectral coherence function SCoh to model our signal and detect the noisy frequencies. To calculate these two functions we used the methods presented by J. Antoni in [16].

## 3. Signal Modelling

### 3.1. Signal Modelling According to Cyclostationarity

Signal modelling is important to know about our system (or our machine). There are different theoretical basis and models of the cyclostationarity, that we can find in [10]. The author cites different modelling types based on the “Amplitude Modulation” Like “Random Amplitude Modulation”, “Bi-component Random Amplitude Modulation”, “Random Amplitude Modulation by a periodic function”, etc.. Then he gives a general model called “Generalized Cyclostationary”.

A stochastic process is generalized cyclostationary when its auto-correlation function is periodic and varying in time. Furthermore, the Fourier coefficients and the cyclic frequencies most be depend on the process delay parameter “τ” (or the frequency “f” in the spectral domain). Hence, we can define the auto-correlation function in this case as:

(7) $$\left[H\right]R_{x}\left(t,\tau \right)=\sum_{n\in I}R_{x}\left(\alpha_{n}\left(\tau \right),\tau \right)e^{j2\pi \alpha_{n}\left(\tau \right)t}$$

with $\alpha_{n}\left(\tau \right)$ is a cyclic frequency depend on τ, *I* is a finite set.

In several application fields, like telecommunication and machine monitoring, systems operate in non-stationary conditions or variable over time. Thus, cyclostationary modulation can be used in many cases. Further, in our case, the turbulent machine generate with a thick aluminium plate ((6) in Figure 3) an airflow in a repetitive way, in other words create a cyclic airflow.

The sound pressure investigated by microphones is the image of this cyclic airflow. That’s why we chose the cyclostationary modulation.

We can model the random process by:

(8) $$X\left(t\right)=U\left(t\right)cos(2\pi f_{r}\left(t\right)t)$$

Or

(9) $$X\left(t\right)=U\left(t\right)sin(2\pi f_{r}\left(t\right)t)$$

with $f_{r}\left(t\right)$ is a variable frequency and U(*t*) is a random signal we will study its case. This model may be exploited in different practical cases, include the cyclostationary ones [10].

The next section develops a tool to extract in real time the evolution of the cyclostationary frequency around 200.8 Hz.

The Section 4 will introduce all details of our experimental setup, divided by 2 sub-sections: Flow configuration and acoustic measurements.

### 3.2. Real Time to Extract Information

From the previous Section 3.1, the information to be extracted is contained in the "frequency" part of the signal (8) or (9). To develop the software sensor, we propose a non-linear algorithm to obtain the frequency information. The technique used was introduced in [17,18]. It is mainly used for signals encountered on electrical domain.

The signal which we propose to reconstruct is described by the function (10):

(10) u(t)=A(t)sin(Φ(t))

Where the total phase Φ(t) can be defined as:

(11) $$\Phi \left(t\right)=\int^{t}\omega \left(\tau \right)d\tau +\delta \left(t\right)$$

The method to extract estimated values of amplitude, frequency and constant phase, respectively $\hat{A}\left(t\right)$, $\hat{\omega }\left(t\right)$, $\hat{\delta }\left(t\right)$, is not developed in this paper. More details are given in [17] and in particular the convergence of the optimization algorithm. The optimization is performed with the help of the gradient descent method.

The result can be summarized by the equations

(12) $$\begin{array}{cc} \frac{d\hat{A}\left(t\right)}{dt} & =2m_{1}e\left(t\right)sin\left(\hat{\phi }\left(t\right)\right), \end{array}$$

(13) $$\begin{array}{cc} \frac{d\hat{\omega }\left(t\right)}{dt} & =2m_{2}e\left(t\right)\hat{A}\left(t\right)cos\left(\hat{\phi }\left(t\right)\right), \end{array}$$

(14) $$\begin{array}{cc} \frac{d\hat{\Phi }\left(t\right)}{dt} & =\hat{\omega }\left(t\right)+m_{3}\frac{d\hat{\omega }\left(t\right)}{dt}, \end{array}$$

with

(15) $$e\left(t\right)=u\left(t\right)-\hat{A}\left(t\right)sin\left(\hat{\phi }\left(t\right)\right)$$

**Figure 1:**
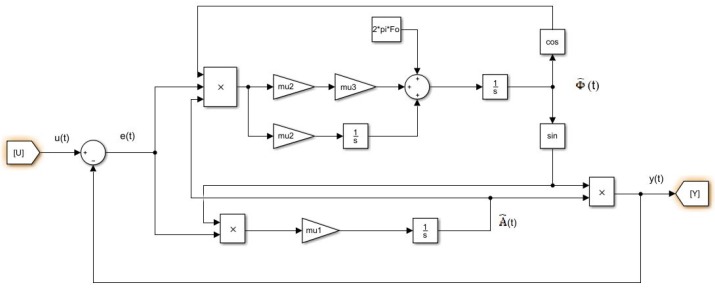
Bloc diagram of the algorithm.

The algorithm is represented by the diagram in the Figure 1.

**Figure 2:**
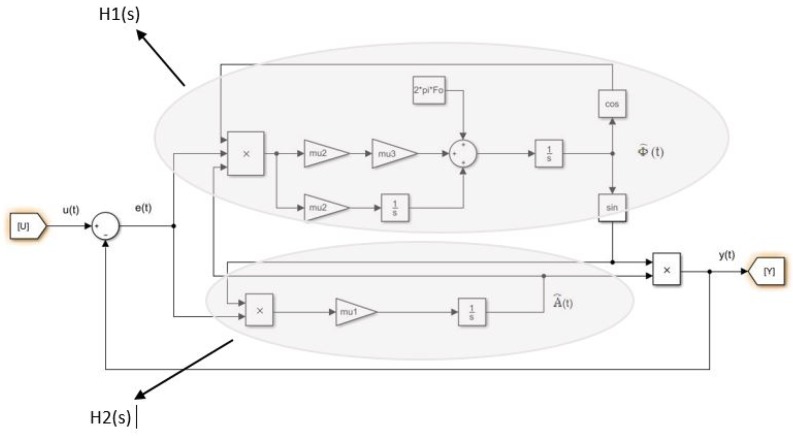
Linearization of the algorithm.

The main difficulty in using this algorithm is its initialization. There is no clear methodology to initialize the $m_{1}$, $m_{2}$ and $m_{3}$ parameters. In this paper, we propose to linearize the algorithm presented (figure) in order to help initialize the parameters. To consider linearization, Figure 2 separates the variations of the signal into two parts: the amplitude part and the frequency part.

The input signal and the the output signal are considered respectively as $u\left(t\right)=\left(A_{0}+\Delta A\left(t\right)\right)sin\left(2\pi \left(f_{0}+\Delta f\left(t\right)\right)t+\phi_{0}\right)$ and $y\left(t\right)=\left(A_{0}+\Delta \hat{A}\left(t\right)\right)sin\left(2\pi \left(f_{0}+\Delta \hat{f}\left(t\right)\right)t+\phi_{0}\right)$.

As shown in Figure 2, after linearization, two transfer functions $H_{1}$ and $H_{2}$ are performed. The linearization makes the coupling disappear and thus during the initialization phase, the estimation of the amplitude can be managed independently of the frequency estimation. The calculations are not detailed in this paper. The result of linearization leads to:

(16) $$H_{1}\left(s\right)=\frac{\Delta \hat{f}\left(s\right)}{\Delta f(s)}=\frac{m_{2}A_{0}^{2}\pi +m_{2}m_{3}A_{0}^{2}\pi s}{s^{2}+m_{2}m_{3}A_{0}^{2}\pi s+m_{2}A_{0}^{2}\pi }$$

(17) $$H_{2}\left(s\right)=\frac{\Delta \hat{A}\left(s\right)}{\Delta A(s)}=\frac{1}{1+\frac{s}{m_{1}}}$$

From simplified models (16) and (17), resulting from linearization, it is possible to adjust $m_{1}$, $m_{2}$ and $m_{3}$ to fix the dynamic performance of the estimator algorithm (magnitude and frequency).

## 4. Experimental Setup

### 4.1. Flow Configuration

**Figure 3:**
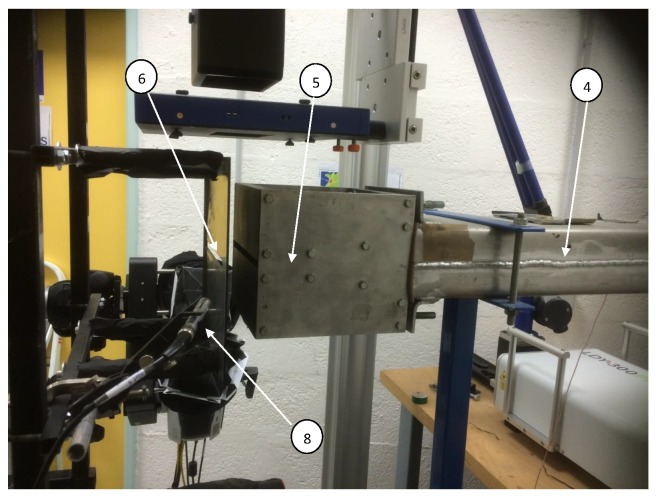
Experimental Set Up.

**Figure 4:**
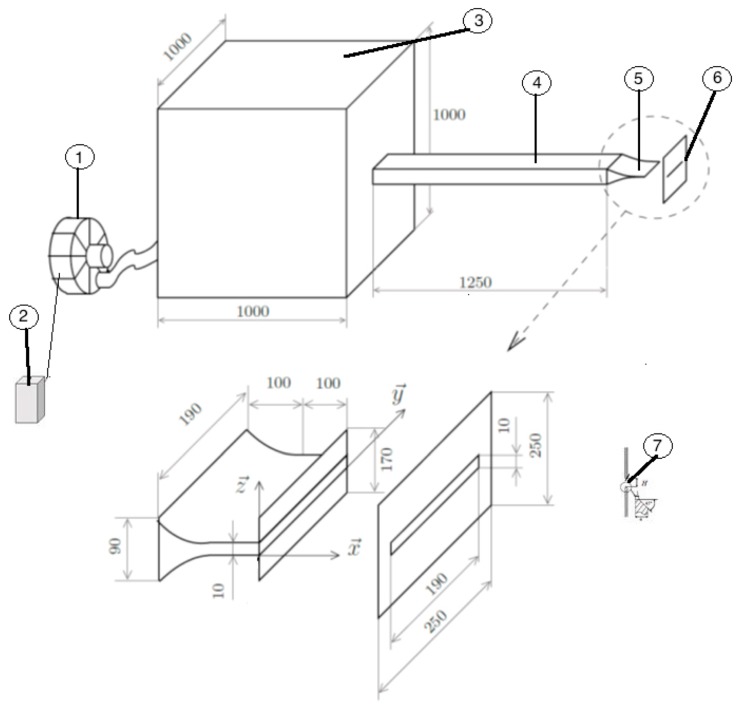
Schematic description of experimental Set Up.

Figure 3 and Figure 4 represent the experimental setup of this study. A controller (2) able to vary the frequency of the compressor (1) responsible for generating the flow. The air generated will be stabilized in a stabilization chamber (3) of 1 m${}^{3}$ before being ejected into a duct of length 1250 mm with a section of 90×200 mm${}^{2}$. Thus, this tube opens out by a convergent of height H=10 mm and of width $L_{z}=200$ mm. In our case, the initial velocity at the exit of the jet is around ($U_{0}=7$ m/s), which corresponds to a Mach number of $M_{0}\approx 0.1$ hence we are in the case of a subsonic jet. Consequently, the flow impacts a plate (6) of thickness 4 mm, having a beveled slot at 45${}^{\circ }$ (7). This split plate is located just in front of the jet outlet and has the same dimensions as the convergent.

The impact distance between the jet outlet and the split plate is indicated by *L*. Thus, in the present study, this impact distance was fixed at 40 mm. Therefore, it was decided to use the dimensionless distance L/H to represent the impact distance. Different parameters are responsible for the variation of Reynolds number ($Re=U_{0}.H/\nu$) such as the kinematic viscosity of the air (ν), the height of the nozzle (*H*) and the speed at the exit of the jet ($U_{0}$). However, during these measurements, the temperature was constant, so the kinematic viscosity did not change, as well as the distance (*H*) is fixed because we are on the same convergent. Thus, the variation of the air speed at the outlet of the jet will be accompanied by an increase in Reynolds number. The work presented in this study was based on a Reynolds number of Re=4458 and at a temperature of 23 ${}^{\circ }C$.

Concerning the structure of the experimental setup, more details are given in [9,19]. The [19] comes from the thesis work [9]. It deals with physical measurements mean to reconstruct a 3D picture of the turbulent flow. Only the flow and its physical characteristics are treated. Our paper, for the experimental part, takes up the experimental setup to develop a software sensor which works in real time and will allow in future works to deal with active control to eliminate the noise nuisance.

### 4.2. Acoustic Measurements

For acoustic treatments, three microphones of type B&K− 4189 were used with a bandwidth between 6.3 Hz and 20,000 Hz. The first was installed behind the slot 8 mm from the plate and far from flow disturbances. The second and third are installed on the wall successively in front of and behind the impact zone between the jet and the split plate. For the acoustic acquisitions, we used a dynamic acquisition card of the national instrument, so we made these acoustic measurements with a frequency of 10,000 Hz for 5 s.

## 5. Experimental Results

**Figure 5:**
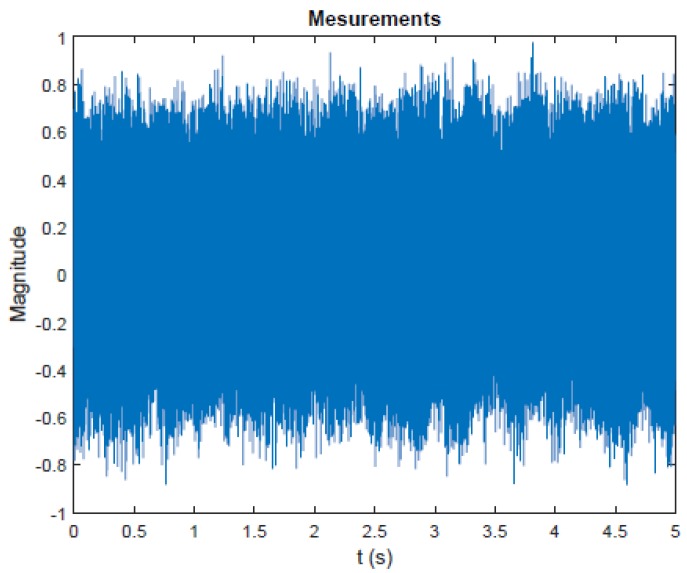
Measurement from microphone (8).

**Figure 6:**
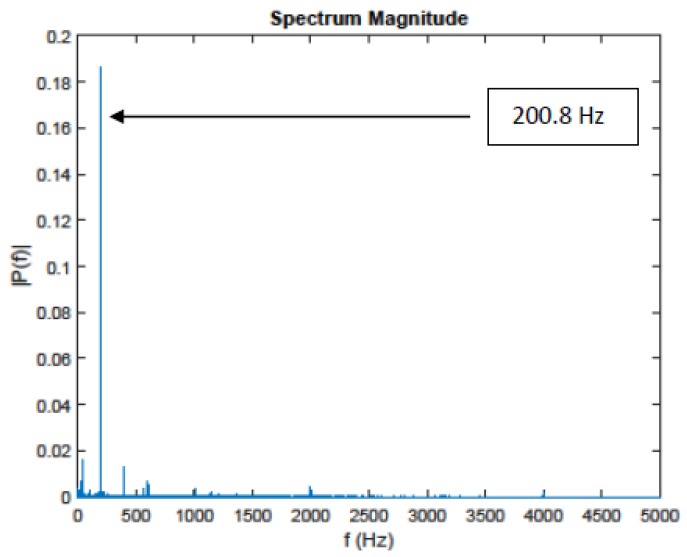
Measurement from microphone (8).

For the processing, we take the recordings obtained from the microphone 8. The Figure 5 plots the measurements in a sampling frequency equal to 10 kHz for a duration of 50,000 samples. With a frequency resolution of Δf = 0.2 Hz, the use of FFT will perform the spectrum of the signal. This spectrum is plotted in the Figure 6.

Every frequency present in the spectrum during the use of the FFT calculation must be stationary. The use of FFT, in the case of the non-stationary signal, provides a result that is not entirely accurate. For our case, over the time horizon of 5 s, the characteristic frequency moves. The value of 200.8 Hz corresponds to a kind of “average”. The proposed method improves its characterization by specifying its properties. This characterization is important in order to then follow the evolution of the characteristic frequency in real time. Hence, for the presence of the non-stationary part of our signal, we chose to apply the cyclo-stationary methods, i.e.,to calculate the cyclic spectral density and the cyclic spectral coherence of the measurements.

**Figure 7:**
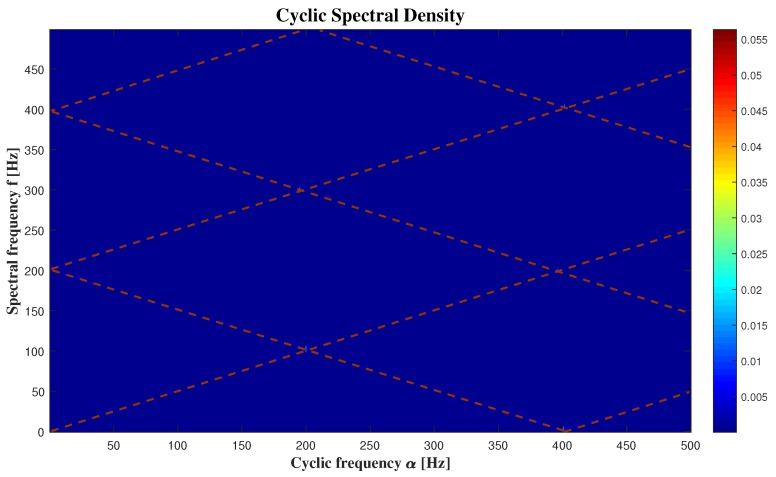
The cyclic spectral density.

**Figure 8:**
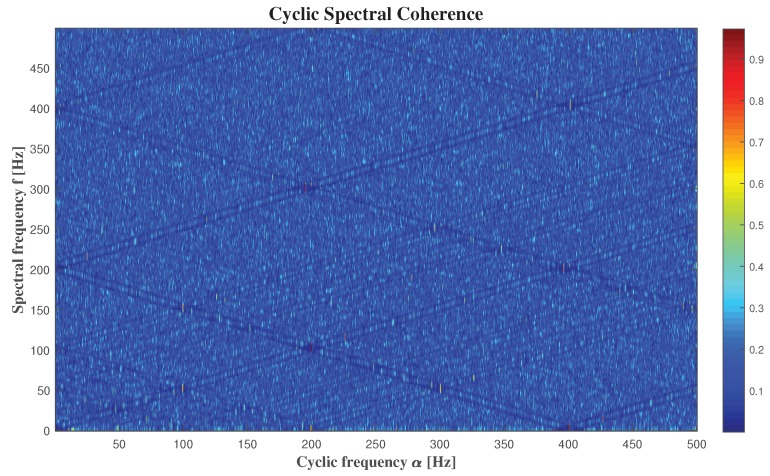
The cyclic spectral coherence.

The Figure 7 presents the Welch’s estimate of the (cross) cyclic power spectrum of the signal coming from microphone 8, and the Figure 8 displays Welch’s estimate of the cyclic spectral coherence of the same signal. We obtain by the two Figure 7 and Figure 8, that the results are the same by using these two methods, but it’s more detailed with the cyclic spectral coherence. That’s why the using of cyclic power spectrum is not enough.

Maiz Sofiane presents in [10] the theoretical relations between the cyclic frequency “α” and the delay parameter “τ”, then he classifies the types of signals based on these relations and the model in Equation (9).

**Figure 9:**
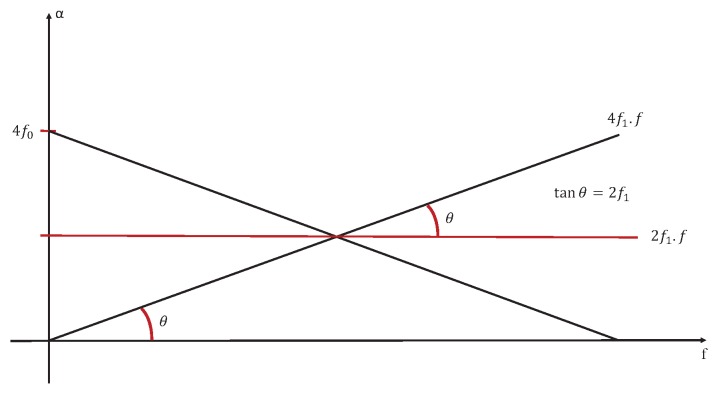
The theoretical relations between “α” and “*f*” for the model (9) when U(t) is stationary.

**Figure 10:**
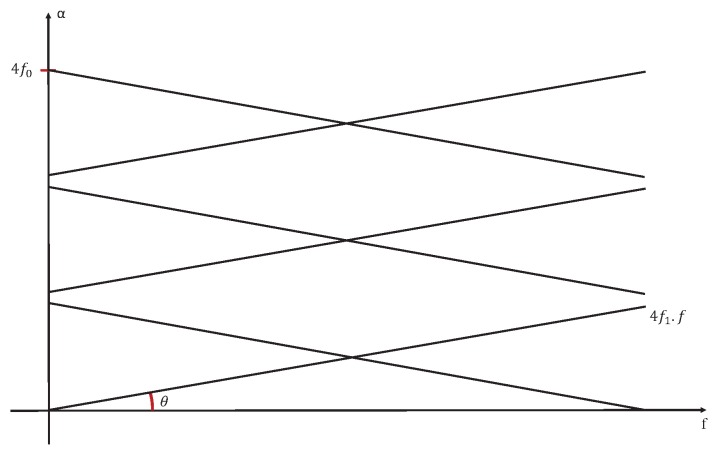
The theoretical relations between “α” and “*f*” for the model (9) when U(t) is cyclostationary.

Based on [10], we can represent the case of signal U(t), when it’s a stationary signal like in Figure 9, or a cyclostationary one as in Figure 10.

However, in our paper, we chose to set the relation between the cyclic frequency “α” and the spectral frequency “*f*” apart from the delay “τ”, when “*f*” is the spectral representation of “τ” in the frequency domain.

We can clearly see that the relation between “α” and “*f*” presented in Figure 7 and Figure 8 is represented by Figure 10. Then we can consider that we have a generalised cyclostationary signal U(t) equal to

$$U\left(t\right)=a\left(t\right)cos\left(2\pi f_{u}t\right)$$

and our model is equal to:

$$X\left(t\right)=U\left(t\right)sin(2\pi f_{r}\left(t\right)t)$$

With 

$f_{r}\left(t\right)=f_{0}+f_{1}t$, 

$f_{U}=200.8$ Hz, 

$f_{0}=125$ Hz, 

$f_{1}=12$ Hz.

When we compare our model to the one in [9], the authors said that the model form is sinusoidal, like ours, which is a good indication of eddies passage.

We can also detect the noisy parts of the audio signals in Figure 7 and Figure 8 by looking at where the frequencies are giving us high intensities. Based on these figures, it is clear that the frequency that gives us the high intensity in 200.8 Hz, is equal to $f_{u}$, and its harmonics. It is expected, because $f_{u}$ is the responsible of the amplitude of our signal.

**Figure 11:**
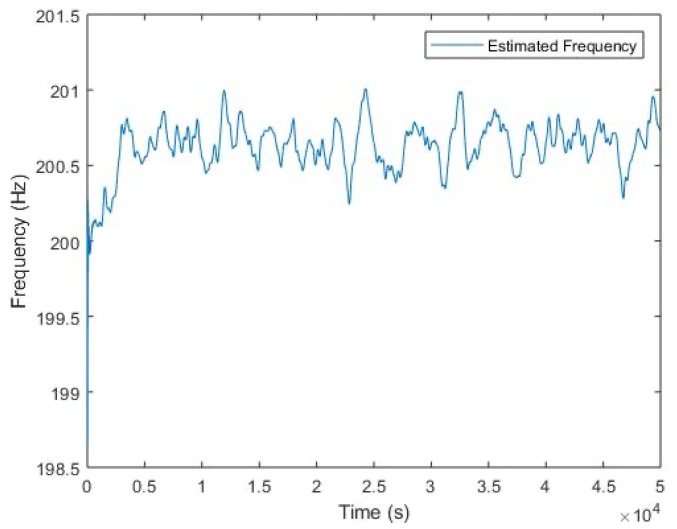
Real time extraction of frequency.

In a real time context, the algorithm presented in Section 3.2 allows to extract the frequency $f_{u}$. The Figure 11 presented the result obtained with real signal.

## 6. Conclusions and Discussion

This paper presents a software sensor development with signal processing tools to model the signals coming from a turbulent machine, and detect the noisy frequencies which are representing physical materials producing the noise. A first analysis based on the use of the FFT tool gives an overall idea of the characteristic frequency. However, this analysis is not sufficient, the physical phenomenon is not stationary. Before proposing a “real-time” monitoring of the characteristic frequency, we propose to demonstrate a property of generalized cyclostationarity of the signal. The use of cyclostationary methods is recommended in this case because of the cyclic airflow created by the turbulent machine. It is thus possible to characterise the experimental setup from the point of view of noise pollution in a precise and dynamic way.

These results have similarity with those in [9] and integration by using software sensor and cyclostationarity tools which are easy to apply and gives good treatment and modelling. We can also use this software sensor to diagnose the machine apart from modelling and noise detection, by other words, for fault detection and perhaps prognosis because of the cyclostationary using.

The algorithm presented in Section 3.2 allows realization of a “real-time” experiential device. To follow a non-stationary frequency that changes around 200 Hz, the sampling frequency is not a technological problem. This software sensor enables tracking a particular frequency dynamically and developing a control law to attenuate the noise nuisance in future work.
